# Hexa­aqua­nickel(II) bis­[tri­aqua-μ_3_-oxalato-di-μ-oxalato-bariumchromate(III)] tetra­hydrate

**DOI:** 10.1107/S2056989020009536

**Published:** 2020-07-17

**Authors:** Yves Alain Mbiangué, Manelsa Lande Ndinga, Jean Pierre Nduga, Emmanuel Wenger, Claude Lecomte

**Affiliations:** a Chemistry Department, Higher Teachers’ Training College, University of Maroua, PO Box 55, Maroua, Cameroon; b Université de Lorraine, CNRS, CRM^2^, F54000, Nancy, France

**Keywords:** crystal structure, tris­(oxalato)chromate(III), barium complexes, nickel complexes, corrugated layers

## Abstract

The title compound, [Ni(H_2_O)_6_][BaCr(C_2_O_4_)_3_(H_2_O)_3_]_2_·4H_2_O, was obtained in the form of single crystals from the slow evaporation of an aqueous mixture of {Ba_6_(H_2_O)_17_[Cr(C_2_O_4_)_3_]_4_}·7H_2_O and NiSO_4_·6H_2_O in the molar ratio 1:4. Its structure is made up of corrugated anionic (101) layers of formula [BaCr(C_2_O_4_)_3_(H_2_O)_3_]*_n_^n^* ^−^ that leave voids accommodating the charge-compensating cations, [Ni(H_2_O)_6_]^2+^ (point group symmetry ), as well as the water mol­ecules of crystallization. The anionic layers are built from the connection of barium and chromium atoms through bridging oxalate ligands. The Cr^III^ atom is hexa­coordinated by O atoms of three oxalate ligands while the Ba^II^ atom is tenfold coordinated by three O atoms of water mol­ecules and seven O atoms of four oxalate ligands. Each Ni^II^ atom sits on an inversion center and is coordinated by six water mol­ecules. One of the uncoordinated water mol­ecules is disordered over two sites, with a refined occupancy ratio of 0.51 (5):0.49 (5). In the crystal, extensive O—H⋯O hydrogen-bonding inter­actions link the anionic layers, the charge-balancing cations as well as the water mol­ecules of crystallization into a three-dimensional supra­molecular network.

## Abstract 2

The structure of the title compound is made up of corrugated anionic layers of formula [BaCr(C_2_O_4_)_3_(H_2_O)_3_]_*n*_^n–^ that leave voids accommodating the charge-compensating cations, [Ni(H_2_O)_6_]^2+^ (point group symmetry 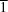), as well as the water mol­ecules of crystallization.

## Chemical context   

Over the past three decades, tris­(oxalato)metalate(III) complex anions, [*M*(C_2_O_4_)_3_]^3–^, have been extensively used for the design of many compounds with fascinating physical properties (Zhong *et al.*, 1990 ▸; Bénard *et al.*, 2001 ▸; Coronado *et al.*, 2008 ▸; Pardo *et al.*, 2011 ▸; Martin *et al.*, 2017 ▸; Tsobnang *et al.*, 2019 ▸; Ōkawa *et al.*, 2020 ▸). One of the main reasons for that is the ability of these anions to act like ligands towards a variety of metallic cations and to build a diversity of extended structures in which neighboring metallic ions are linked through bridging oxalate ligands. From the synthetic point of view, the tris­(oxalato)chromate(III) anion, [Cr(C_2_O_4_)_3_]^3–^ or [Cr(ox)_3_]^3–^, is most attractive because of its stability and inertness toward ligand substitution. As a source of this anion, the polymeric complex salt {Ba_6_(H_2_O)_17_[Cr(C_2_O_4_)_3_]_4_}·7H_2_O (Bélombé *et al.*, 2003 ▸) offers the possibility of easily replacing, in the reaction medium and under daylight, the Ba^2+^ ions by other cations, provided the latter are brought into that medium as their sulfates. Since Ba^2+^ has a flexible coordination sphere with coordination numbers ranging from three to twelve (Hancock *et al.*, 2004 ▸), this inspired us to start a research program aimed at exploring the various structures that might arise from different combinations of [Cr(ox)_3_]^3–^, Ba^2+^ and other cations, and possibly studying the physical properties of the corresponding compounds. From an aqueous suspension of {Ba_6_(H_2_O)_17_[Cr(C_2_O_4_)_3_]_4_}·7H_2_O, a partial replacement of Ba^2+^ by Ni^2+^ led to [Ni(H_2_O)_6_][BaCr(C_2_O_4_)_3_(H_2_O)_3_]_2_·4H_2_O (**I**), the structure of which is described herein.
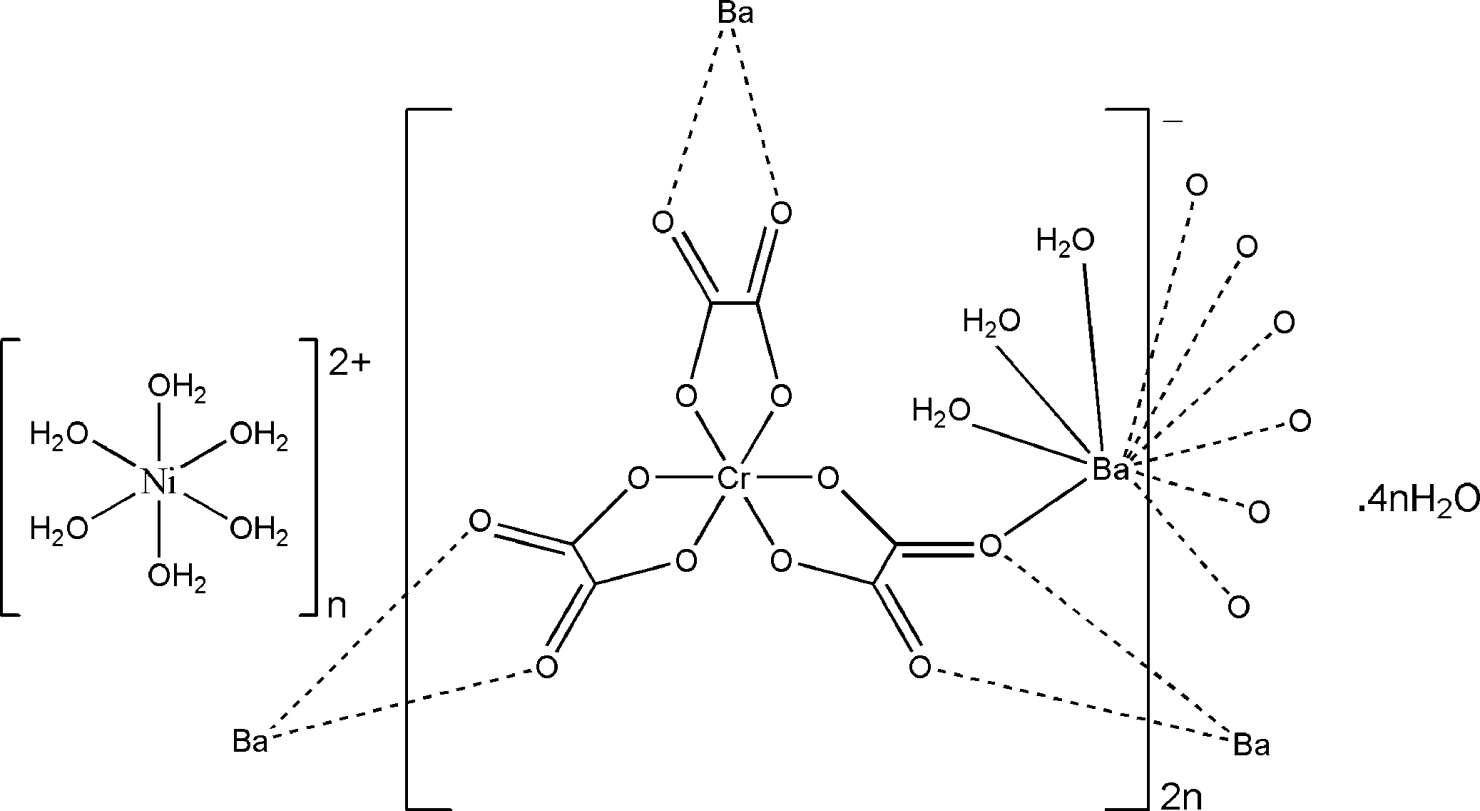


## Structural commentary   

**Figure 1:**
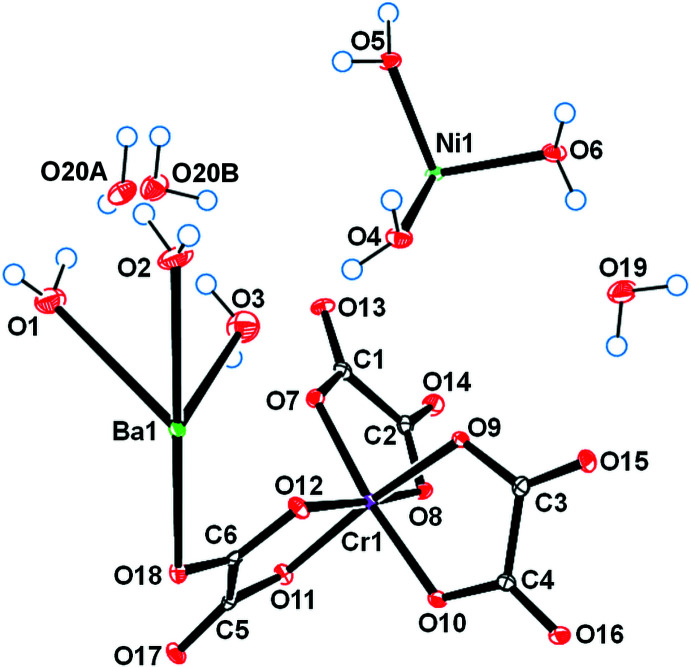
The components of the asymmetric unit of (I), showing the atom-numbering scheme and displacement ellipsoids at the 50% probability level.

**Figure 2:**
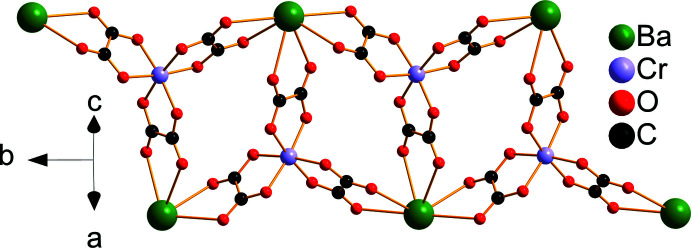
Connection of [Cr(C_2_O_4_)_3_]^3–^ units with Ba^2+^ cations into a ladder-like chain. Barium-coordinating water mol­ecules have been omitted for clarity.

**Figure 3:**
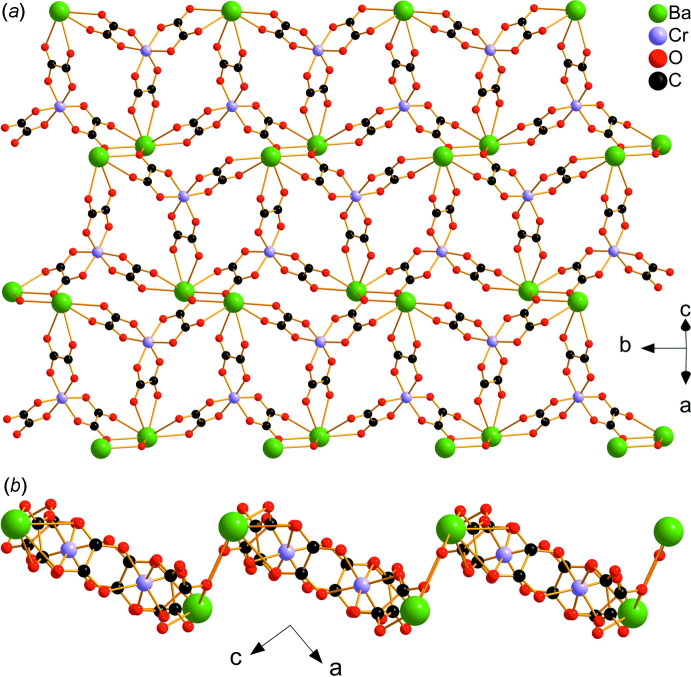
Three adjacent ladder-like chains connected through Ba_2_O_2_ units into a corrugated layer, viewed in the (101) plane (*a*) and along [010] (*b*). Barium-coordinating water mol­ecules have been omitted for clarity.

**Figure 4:**
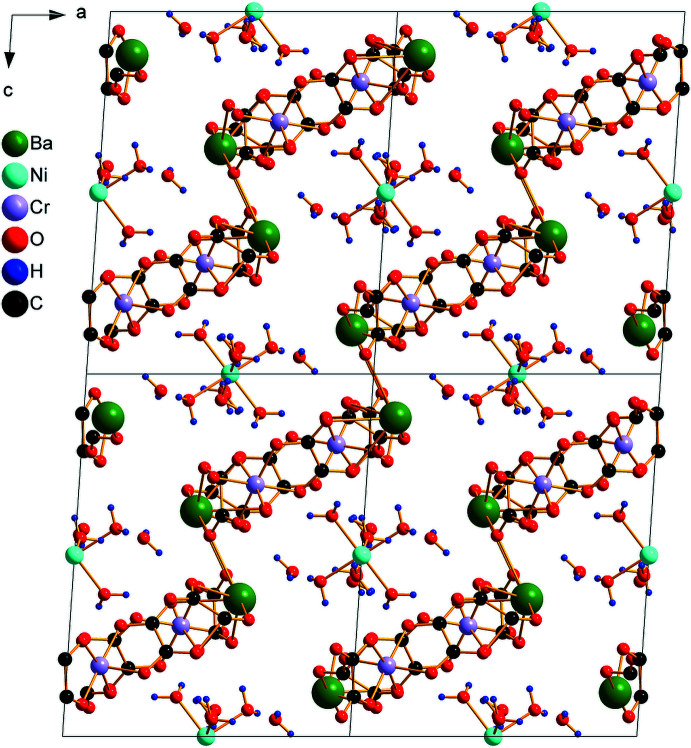
Packing of the crystal structure of (I) in a view along [010], showing corrugated layers inter­leaved by [Ni(H_2_O)_6_]^2+^ complex cations and water mol­ecules of crystallization. Barium-coordinating water mol­ecules have been omitted for clarity.

The asymmetric unit of (**I**) is depicted in Fig. 1 ▸. It contains one half of an [Ni(H_2_O)_6_]^2+^ cation situated on an inversion center, one [BaCr(C_2_O_4_)_3_(H_2_O)_3_]^−^ anion and two water mol­ecules of crystallization, one of which being equally disordered over two positions (O20*A* and O20*B*). The Ba^2+^ ion is linked to ten O atoms from three water mol­ecules and four oxalate ligands (three chelating, one monodentately binding), with Ba—O bond lengths in the range 2.784 (3)–2.933 (3) Å (Table 1 ▸). These values are typical for ten-coordinate barium complexes with oxalate and water ligands (Alabada *et al.*, 2015 ▸). One of the oxalate ligands (bearing O18) bridges three cations (two Ba and one Cr) while the two others are bis-chelating (one Ba and one Cr). In the crystal, neighboring [Cr(C_2_O_4_)_3_]^3–^ units are linked through barium ions into a ladder-like chain running parallel to [010] (Fig. 2 ▸). Adjacent ladders are then connected, through Ba—O18 coordination bonds, into a corrugated layer extending parallel to (101) (Fig. 3 ▸). The packing of the layers delineates voids that accommodate the cationic complex, [Ni(H_2_O)_6_]^2+^, as well as the water mol­ecules of crystallization (Fig. 4 ▸).

## Supra­molecular features   

In the crystal, extensive O—H⋯O hydrogen-bonding inter­actions of medium-to-weak strength are observed (Table 2 ▸), with all the water mol­ecules acting as hydrogen-bond donors. The water mol­ecules of crystallization also act as hydrogen-bond acceptors, as well as all of the oxalate O atoms except O12, O14 and O18. Two barium-coordinating water mol­ecules (O1 and O3) behave as hydrogen-bond donors toward both components of the disordered lattice water mol­ecule (O20*A* and O20*B*) *via* three-center bonds, O1—H1*B*⋯(O20*A*,O20*B*) and O3—H3*B*⋯(O20*A*,O20*B*). The cationic complex, [Ni(H_2_O)_6_]^2+^, functions as a hydrogen-bond donor group towards one barium-coordinating water mol­ecule (O3), one water mol­ecule of crystallization (O19) and four oxalate O atoms, *viz*. O9^vi^, O13^vi^, O11^iv^ and O17^iv^ [symmetry codes refer to Table 2 ▸]. Together, these inter­actions lead to a three-dimensional supra­molecular network structure.

## Database survey   

A search of the Cambridge Structural Database (CSD version 5.41, May 2020; Groom *et al.*, 2016 ▸) for [*M*(C_2_O_4_)_3_]^*n*−^ complexes with each oxalate ligand bis-chelating *M* and another metal *M*′ gave 316 hits. Of these hits, 86 contain *M* = Cr and only one, the parent complex of (I), contains *M* = Cr and *M*′ = Ba.

## Synthesis and crystallization   

The parent complex of (I), {Ba_6_(H_2_O)_17_[Cr(C_2_O_4_)_3_]_4_}·7H_2_O, was prepared as previously described (Bélombé *et al.*, 2003 ▸). The title compound was synthesized as follows: NiSO_4_·6H_2_O (0.21 g, 0.8 mmol) was dissolved in water (20 ml) and the resulting green solution added dropwise, under stirring and at 313 K, to a violet suspension of {Ba_6_(H_2_O)_17_[Cr(C_2_O_4_)_3_]_4_}·7H_2_O (0.50 g, 0.2 mmol) in water (25 ml). After one h, the colorless precipitate of BaSO_4_ was filtered off, and the filtrate was left to evaporate at room temperature. Two days later, crystals suitable for X-ray analysis were harvested.

## Refinement   

Crystal data, data collection and structure refinement details are summarized in Table 3 ▸. All hydrogen atoms were located in difference-Fourier maps and refined with O—H and H⋯H distance restraints of 0.88 (1) and 1.37 (2) Å, respectively, and with *U*_iso_(H) = 1.5*U*_eq_(O). One lattice water mol­ecule was refined as being disordered over two positions (O20*A* and O20*B*), with the occupancy ratio refined to 0.51 (5):0.49 (5). The distances Ba1—H3*A* and Ba1—H3*B* were restrained to be equal using a SADI instruction.

## Supplementary Material

Crystal structure: contains datablock(s) I. DOI: 10.1107/S2056989020009536/wm5572sup1.cif

Structure factors: contains datablock(s) I. DOI: 10.1107/S2056989020009536/wm5572Isup2.hkl

CCDC reference: 1953456

Additional supporting information:  crystallographic information; 3D view; checkCIF report

## Figures and Tables

**Table 1:** Selected bond lengths (Å)

Ba1—O2	2.784 (3)	Ba1—O18^i^	2.873 (2)
Ba1—O17^i^	2.802 (2)	Ba1—O13^iii^	2.874 (2)
Ba1—O15^ii^	2.855 (2)	Ba1—O3	2.880 (3)
Ba1—O18	2.856 (2)	Ba1—O14^iii^	2.912 (2)
Ba1—O16^ii^	2.859 (2)	Ba1—O1	2.933 (2)

Symmetry codes: (i) 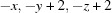; (ii) 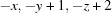; (iii) 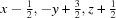.

**Table 2:** Hydrogen-bond geometry (Å, °)

*D*—H⋯*A*	*D*—H	H⋯*A*	*D*⋯*A*	*D*—H⋯*A*
O1—H1*A*⋯O16^iv^	0.88 (1)	2.20 (2)	3.038 (4)	160 (3)
O1—H1*B*⋯O20*A*	0.88 (1)	1.80 (2)	2.660 (18)	165 (4)
O1—H1*B*⋯O20*B*	0.88 (1)	2.25 (3)	3.08 (2)	156 (3)
O2—H2*A*⋯O10^iv^	0.88 (1)	1.98 (1)	2.851 (3)	175 (4)
O2—H2*B*⋯O19^v^	0.88 (1)	1.96 (1)	2.835 (4)	175 (4)
O3—H3*A*⋯O7	0.89 (1)	2.32 (3)	2.993 (3)	132 (3)
O3—H3*B*⋯O20*A*	0.89 (1)	2.00 (2)	2.893 (16)	176 (3)
O3—H3*B*⋯O20*B*	0.89 (1)	1.86 (2)	2.722 (11)	163 (3)
O4—H4*A*⋯O3	0.88 (1)	1.96 (1)	2.819 (4)	166 (4)
O4—H4*B*⋯O17^iv^	0.87 (1)	1.92 (1)	2.791 (3)	179 (4)
O5—H5*A*⋯O11^iv^	0.87 (1)	1.96 (1)	2.811 (3)	165 (4)
O5—H5*B*⋯O9^vi^	0.88 (1)	1.84 (1)	2.703 (3)	167 (4)
O6—H6*A*⋯O13^vi^	0.87 (1)	1.91 (1)	2.761 (3)	165 (4)
O6—H6*B*⋯O19	0.87 (1)	1.83 (1)	2.693 (3)	172 (3)
O19—H19*A*⋯O1^vii^	0.87 (1)	1.94 (2)	2.759 (4)	157 (4)
O19—H19*B*⋯O15	0.87 (1)	1.93 (1)	2.789 (3)	172 (4)
O20*A*—H20*A*⋯O8^iv^	0.88 (1)	2.01 (3)	2.855 (10)	161 (8)
O20*A*—H20*B*⋯O20*A* ^viii^	0.88 (1)	1.74 (3)	2.60 (2)	168 (9)
O20*B*—H20*C*⋯O8^iv^	0.88 (1)	2.11 (4)	2.893 (11)	149 (7)
O20*B*—H20*D*⋯O6^vi^	0.88 (1)	2.07 (4)	2.90 (3)	159 (8)

Symmetry codes: (iv) 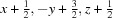; (v) 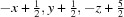; (vi) 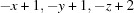; (vii) 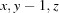; (viii) 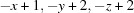.

**Table 3:** Experimental details

Crystal data	
Chemical formula	[Ni(H_2_O)_6_][BaCr(C_2_O_4_)_3_(H_2_O)_3_]_2_·4H_2_O
*M*_r_	1253.76
Crystal system, space group	Monoclinic, *P*2_1_/*n*
Temperature (K)	100
*a*, *b*, *c* (Å)	11.5556 (11), 11.0774 (13), 14.6105 (17)
β (°)	93.794 (4)
*V* (Å^3^)	1866.1 (4)
*Z*	2
Radiation type	Mo *K*α
μ (mm^−1^)	3.27
Crystal size (mm)	0.14 × 0.09 × 0.06

Data collection	
Diffractometer	Bruker D8 Venture
Absorption correction	Multi-scan (*SADABS*; Krause *et al.*, 2015 ▸)
*T*_min_, *T*_max_	0.564, 0.746
No. of measured, independent and observed [*I* > 2σ(*I*)] reflections	53111, 4278, 3539
*R*_int_	0.102
(sin θ/λ)_max_ (Å^−1^)	0.650

Refinement	
*R*[*F* ^2^ > 2σ(*F* ^2^)], *wR*(*F* ^2^), *S*	0.026, 0.058, 1.03
No. of reflections	4278
No. of parameters	324
No. of restraints	28
H-atom treatment	Only H-atom coordinates refined
Δρ_max_, Δρ_min_ (e Å^−3^)	0.91, −0.85

Computer programs: *APEX2* and *SAINT* (Bruker, 2012 ▸), *SHELXS* (Sheldrick, 2015*a* ▸), *SHELXL2018/3* (Sheldrick, 2015*b* ▸), *ORTEP-3 for Windows* and *WinGX* (Farrugia, 2012 ▸), *DIAMOND* (Brandenburg & Putz, 2018 ▸) and *publCIF* (Westrip, 2010 ▸).

## supplementary crystallographic information

### Crystal data

**Table:**

[Ni(H_2_O)_6_][BaCr(C_2_O_4_)_3_(H_2_O)_3_]_2_·4H_2_O	*F*(000) = 1224
*M**_r_* = 1253.76	*D*_x_ = 2.231 Mg m^−^^3^
Monoclinic, *P*2_1_/*n*	Mo *K*α radiation, λ = 0.71073 Å
*a* = 11.5556 (11) Å	Cell parameters from 53111 reflections
*b* = 11.0774 (13) Å	θ = 2.2–27.5°
*c* = 14.6105 (17) Å	µ = 3.27 mm^−^^1^
β = 93.794 (4)°	*T* = 100 K
*V* = 1866.1 (4) Å^3^	Block, metallic dark red
*Z* = 2	0.14 × 0.09 × 0.06 mm

### Data collection

**Table:**

Bruker D8 Venture diffractometer	3539 reflections with *I* > 2σ(*I*)
ω scans	*R*_int_ = 0.102
Absorption correction: multi-scan (*SADABS*; Krause *et al.*, 2015)	θ_max_ = 27.5°, θ_min_ = 2.2°
*T*_min_ = 0.564, *T*_max_ = 0.746	*h* = −15→14
53111 measured reflections	*k* = −14→14
4278 independent reflections	*l* = −18→18

### Refinement

**Table:**

Refinement on *F*^2^	Secondary atom site location: difference Fourier map
Least-squares matrix: full	Hydrogen site location: difference Fourier map
*R*[*F*^2^ > 2σ(*F*^2^)] = 0.026	Only H-atom coordinates refined
*wR*(*F*^2^) = 0.058	*w* = 1/[σ^2^(*F*_o_^2^) + (0.0172*P*)^2^ + 3.0153*P*] where *P* = (*F*_o_^2^ + 2*F*_c_^2^)/3
*S* = 1.03	(Δ/σ)_max_ = 0.001
4278 reflections	Δρ_max_ = 0.91 e Å^−^^3^
324 parameters	Δρ_min_ = −0.85 e Å^−^^3^
28 restraints	Extinction correction: SHELXL-2018/3 (Sheldrick 2015b), Fc^*^=kFc[1+0.001xFc^2^λ^3^/sin(2θ)]^-1/4^
Primary atom site location: structure-invariant direct methods	Extinction coefficient: 0.00113 (15)

### Special details

**Table:**

Geometry. All esds (except the esd in the dihedral angle between two l.s. planes) are estimated using the full covariance matrix. The cell esds are taken into account individually in the estimation of esds in distances, angles and torsion angles; correlations between esds in cell parameters are only used when they are defined by crystal symmetry. An approximate (isotropic) treatment of cell esds is used for estimating esds involving l.s. planes.

### Fractional atomic coordinates and isotropic or equivalent isotropic displacement parameters (Å^2^)

**Table:**

	*x*	*y*	*z*	*U*_iso_*/*U*_eq_	Occ. (<1)
Ba1	0.08524 (2)	0.86116 (2)	1.12114 (2)	0.00717 (7)	
Ni1	0.500000	0.500000	1.000000	0.00719 (12)	
Cr1	0.11343 (4)	0.64371 (4)	0.80510 (3)	0.00671 (11)	
O1	0.2825 (2)	1.0283 (2)	1.13529 (18)	0.0194 (5)	
H1A	0.295 (3)	1.065 (4)	1.1884 (15)	0.029*	
H1B	0.3528 (16)	1.008 (4)	1.120 (3)	0.029*	
O2	0.2458 (2)	0.8272 (2)	1.2695 (2)	0.0254 (6)	
H2A	0.311 (2)	0.867 (4)	1.273 (3)	0.038*	
H2B	0.242 (3)	0.798 (4)	1.3252 (14)	0.038*	
O3	0.2799 (2)	0.7817 (2)	1.02345 (18)	0.0239 (6)	
H3A	0.268 (3)	0.809 (4)	0.9661 (11)	0.036*	
H3B	0.340 (2)	0.829 (3)	1.041 (2)	0.036*	
O4	0.36196 (19)	0.5495 (2)	1.07281 (16)	0.0128 (5)	
H4A	0.327 (3)	0.6188 (19)	1.064 (2)	0.019*	
H4B	0.384 (3)	0.551 (3)	1.1313 (9)	0.019*	
O5	0.61283 (18)	0.5438 (2)	1.10821 (15)	0.0111 (5)	
H5A	0.597 (3)	0.606 (2)	1.141 (2)	0.017*	
H5B	0.6886 (9)	0.541 (3)	1.109 (3)	0.017*	
O6	0.4836 (2)	0.3258 (2)	1.04722 (16)	0.0117 (5)	
H6A	0.508 (3)	0.304 (3)	1.1027 (12)	0.018*	
H6B	0.4116 (13)	0.301 (3)	1.041 (2)	0.018*	
O7	0.27008 (18)	0.71183 (19)	0.82518 (14)	0.0088 (4)	
O8	0.17713 (18)	0.57697 (19)	0.69450 (15)	0.0098 (4)	
O9	0.15646 (18)	0.49939 (19)	0.87894 (15)	0.0095 (4)	
O10	−0.03403 (18)	0.55595 (19)	0.78547 (15)	0.0105 (5)	
O11	0.04723 (18)	0.78461 (19)	0.73672 (14)	0.0090 (4)	
O12	0.06071 (18)	0.73167 (19)	0.91145 (15)	0.0099 (4)	
O13	0.44555 (18)	0.7013 (2)	0.76971 (15)	0.0109 (5)	
O14	0.34645 (19)	0.5579 (2)	0.62938 (16)	0.0125 (5)	
O15	0.08516 (19)	0.32087 (19)	0.92016 (15)	0.0117 (5)	
O16	−0.12046 (19)	0.3838 (2)	0.82179 (17)	0.0158 (5)	
O17	−0.06735 (19)	0.94310 (19)	0.75914 (15)	0.0103 (4)	
O18	−0.03303 (18)	0.89973 (19)	0.94573 (15)	0.0088 (4)	
O19	0.2628 (2)	0.2480 (2)	1.04657 (19)	0.0244 (6)	
H19A	0.256 (4)	0.1727 (15)	1.062 (3)	0.037*	
H19B	0.206 (3)	0.263 (3)	1.007 (2)	0.037*	
O20A	0.4749 (9)	0.938 (2)	1.0718 (8)	0.015 (3)	0.51 (5)
H20A	0.543 (3)	0.923 (8)	1.099 (5)	0.023*	0.51 (5)
H20B	0.493 (6)	0.970 (8)	1.020 (3)	0.023*	0.51 (5)
O20B	0.4877 (12)	0.895 (2)	1.0565 (11)	0.018 (2)	0.49 (5)
H20C	0.536 (6)	0.875 (7)	1.103 (4)	0.027*	0.49 (5)
H20D	0.477 (7)	0.825 (4)	1.028 (5)	0.027*	0.49 (5)
C1	0.3415 (3)	0.6770 (3)	0.7665 (2)	0.0085 (6)	
C2	0.2869 (3)	0.5965 (3)	0.6882 (2)	0.0087 (6)	
C3	0.0763 (3)	0.4184 (3)	0.8807 (2)	0.0081 (6)	
C4	−0.0373 (3)	0.4528 (3)	0.8258 (2)	0.0092 (6)	
C5	−0.0088 (3)	0.8571 (3)	0.7871 (2)	0.0073 (6)	
C6	0.0054 (3)	0.8296 (3)	0.8905 (2)	0.0078 (6)	

### Atomic displacement parameters (Å^2^)

**Table:**

	*U*^11^	*U*^22^	*U*^33^	*U*^12^	*U*^13^	*U*^23^
Ba1	0.00780 (10)	0.00561 (9)	0.00811 (10)	0.00062 (7)	0.00049 (6)	0.00064 (7)
Ni1	0.0055 (3)	0.0091 (3)	0.0068 (3)	0.0002 (2)	−0.0008 (2)	−0.0013 (2)
Cr1	0.0055 (2)	0.0055 (2)	0.0092 (2)	0.00058 (18)	0.00116 (18)	0.00096 (19)
O1	0.0149 (12)	0.0216 (13)	0.0213 (14)	−0.0025 (10)	−0.0011 (10)	0.0013 (11)
O2	0.0171 (13)	0.0242 (14)	0.0329 (16)	−0.0095 (11)	−0.0126 (12)	0.0088 (12)
O3	0.0239 (14)	0.0266 (15)	0.0211 (14)	0.0006 (11)	0.0012 (11)	−0.0033 (11)
O4	0.0101 (11)	0.0168 (12)	0.0113 (11)	0.0026 (9)	0.0003 (9)	−0.0013 (9)
O5	0.0063 (10)	0.0142 (11)	0.0127 (12)	0.0021 (9)	−0.0008 (9)	−0.0051 (9)
O6	0.0103 (11)	0.0144 (11)	0.0100 (11)	0.0000 (9)	−0.0020 (9)	0.0033 (9)
O7	0.0073 (10)	0.0086 (10)	0.0105 (11)	−0.0005 (8)	0.0015 (8)	−0.0015 (9)
O8	0.0063 (10)	0.0120 (11)	0.0111 (11)	0.0000 (8)	0.0010 (8)	−0.0022 (9)
O9	0.0063 (10)	0.0064 (10)	0.0155 (12)	0.0000 (8)	−0.0014 (9)	0.0020 (9)
O10	0.0066 (10)	0.0087 (10)	0.0162 (12)	0.0000 (8)	−0.0004 (9)	0.0035 (9)
O11	0.0101 (10)	0.0086 (10)	0.0084 (11)	0.0011 (8)	0.0024 (8)	0.0010 (9)
O12	0.0114 (11)	0.0095 (11)	0.0088 (11)	0.0028 (9)	0.0017 (8)	0.0033 (9)
O13	0.0061 (10)	0.0157 (12)	0.0108 (11)	−0.0021 (8)	0.0002 (8)	0.0009 (9)
O14	0.0097 (11)	0.0139 (11)	0.0142 (12)	−0.0003 (9)	0.0042 (9)	−0.0018 (9)
O15	0.0144 (11)	0.0076 (10)	0.0126 (12)	−0.0002 (9)	−0.0019 (9)	0.0036 (9)
O16	0.0075 (11)	0.0110 (12)	0.0282 (14)	−0.0014 (9)	−0.0034 (10)	0.0037 (10)
O17	0.0138 (11)	0.0086 (11)	0.0081 (11)	0.0038 (9)	−0.0018 (9)	0.0014 (9)
O18	0.0117 (11)	0.0070 (10)	0.0078 (11)	−0.0003 (8)	0.0017 (9)	0.0000 (8)
O19	0.0201 (13)	0.0203 (14)	0.0307 (15)	−0.0076 (11)	−0.0138 (11)	0.0119 (12)
O20A	0.015 (3)	0.021 (7)	0.009 (4)	−0.003 (4)	0.000 (2)	−0.004 (4)
O20B	0.021 (4)	0.020 (6)	0.012 (4)	−0.003 (4)	−0.002 (3)	−0.001 (4)
C1	0.0100 (15)	0.0081 (13)	0.0075 (15)	0.0006 (11)	0.0005 (12)	0.0030 (11)
C2	0.0106 (15)	0.0068 (13)	0.0089 (15)	−0.0004 (11)	0.0010 (12)	0.0019 (12)
C3	0.0093 (14)	0.0079 (15)	0.0074 (15)	0.0000 (12)	0.0021 (12)	−0.0029 (12)
C4	0.0066 (14)	0.0085 (14)	0.0124 (16)	0.0011 (11)	0.0010 (12)	−0.0007 (12)
C5	0.0071 (14)	0.0067 (13)	0.0081 (14)	−0.0027 (12)	0.0014 (11)	−0.0009 (12)
C6	0.0048 (14)	0.0070 (14)	0.0115 (15)	−0.0035 (11)	−0.0005 (12)	0.0026 (12)

### Geometric parameters (Å, º)

**Table:**

Ba1—O2	2.784 (3)	O4—H4A	0.875 (10)
Ba1—O17^i^	2.802 (2)	O4—H4B	0.874 (10)
Ba1—O15^ii^	2.855 (2)	O5—H5A	0.870 (10)
Ba1—O18	2.856 (2)	O5—H5B	0.875 (10)
Ba1—O16^ii^	2.859 (2)	O6—H6A	0.873 (10)
Ba1—O18^i^	2.873 (2)	O6—H6B	0.874 (10)
Ba1—O13^iii^	2.874 (2)	O7—C1	1.288 (4)
Ba1—O3	2.880 (3)	O8—C2	1.297 (4)
Ba1—O14^iii^	2.912 (2)	O9—C3	1.291 (4)
Ba1—O1	2.933 (2)	O10—C4	1.287 (4)
Ni1—O5^iv^	2.040 (2)	O11—C5	1.292 (4)
Ni1—O5	2.040 (2)	O12—C6	1.285 (4)
Ni1—O4^iv^	2.050 (2)	O13—C1	1.230 (4)
Ni1—O4	2.050 (2)	O14—C2	1.213 (4)
Ni1—O6^iv^	2.062 (2)	O15—C3	1.225 (4)
Ni1—O6	2.062 (2)	O16—C4	1.226 (4)
Cr1—O8	1.964 (2)	O17—C5	1.223 (4)
Cr1—O12	1.965 (2)	O18—C6	1.225 (4)
Cr1—O7	1.965 (2)	O19—H19A	0.870 (10)
Cr1—O10	1.966 (2)	O19—H19B	0.870 (10)
Cr1—O9	1.974 (2)	O20A—H20A	0.878 (10)
Cr1—O11	1.979 (2)	O20A—H20B	0.878 (10)
O1—H1A	0.879 (10)	O20B—H20C	0.878 (10)
O1—H1B	0.884 (10)	O20B—H20D	0.879 (10)
O2—H2A	0.875 (10)	C1—C2	1.550 (4)
O2—H2B	0.878 (10)	C3—C4	1.542 (4)
O3—H3A	0.893 (10)	C5—C6	1.540 (4)
O3—H3B	0.891 (10)		
			
O2—Ba1—O17^i^	72.03 (7)	O12—Cr1—O10	92.84 (9)
O2—Ba1—O15^ii^	118.93 (7)	O7—Cr1—O10	172.94 (9)
O17^i^—Ba1—O15^ii^	126.88 (6)	O8—Cr1—O9	92.88 (9)
O2—Ba1—O18	166.84 (7)	O12—Cr1—O9	92.84 (9)
O17^i^—Ba1—O18	113.19 (6)	O7—Cr1—O9	91.90 (9)
O15^ii^—Ba1—O18	68.50 (6)	O10—Cr1—O9	82.17 (9)
O2—Ba1—O16^ii^	64.53 (7)	O8—Cr1—O11	92.00 (9)
O17^i^—Ba1—O16^ii^	124.55 (7)	O12—Cr1—O11	83.02 (9)
O15^ii^—Ba1—O16^ii^	58.29 (6)	O7—Cr1—O11	95.39 (9)
O18—Ba1—O16^ii^	117.00 (7)	O10—Cr1—O11	90.80 (9)
O2—Ba1—O18^i^	120.24 (7)	O9—Cr1—O11	171.68 (9)
O17^i^—Ba1—O18^i^	58.43 (6)	Ba1—O1—H1A	116 (3)
O15^ii^—Ba1—O18^i^	116.93 (6)	Ba1—O1—H1B	123 (3)
O18—Ba1—O18^i^	58.65 (7)	H1A—O1—H1B	104 (2)
O16^ii^—Ba1—O18^i^	175.13 (6)	Ba1—O2—H2A	121 (3)
O2—Ba1—O13^iii^	76.00 (7)	Ba1—O2—H2B	134 (3)
O17^i^—Ba1—O13^iii^	69.29 (6)	H2A—O2—H2B	103 (2)
O15^ii^—Ba1—O13^iii^	64.89 (6)	Ba1—O3—H3A	106 (2)
O18—Ba1—O13^iii^	117.03 (6)	Ba1—O3—H3B	107 (2)
O16^ii^—Ba1—O13^iii^	68.14 (7)	H3A—O3—H3B	99 (2)
O18^i^—Ba1—O13^iii^	111.27 (6)	Ni1—O4—H4A	122 (3)
O2—Ba1—O3	80.94 (8)	Ni1—O4—H4B	109 (2)
O17^i^—Ba1—O3	129.65 (7)	H4A—O4—H4B	103 (2)
O15^ii^—Ba1—O3	103.29 (7)	Ni1—O5—H5A	118 (2)
O18—Ba1—O3	86.79 (7)	Ni1—O5—H5B	126 (2)
O16^ii^—Ba1—O3	75.75 (7)	H5A—O5—H5B	105 (2)
O18^i^—Ba1—O3	105.45 (7)	Ni1—O6—H6A	123 (2)
O13^iii^—Ba1—O3	142.83 (7)	Ni1—O6—H6B	111 (2)
O2—Ba1—O14^iii^	126.45 (8)	H6A—O6—H6B	104 (2)
O17^i^—Ba1—O14^iii^	68.07 (6)	C1—O7—Cr1	114.17 (19)
O15^ii^—Ba1—O14^iii^	65.42 (6)	C2—O8—Cr1	114.68 (19)
O18—Ba1—O14^iii^	65.95 (6)	C3—O9—Cr1	114.79 (19)
O16^ii^—Ba1—O14^iii^	113.40 (6)	C4—O10—Cr1	115.08 (19)
O18^i^—Ba1—O14^iii^	63.38 (6)	C5—O11—Cr1	113.24 (19)
O13^iii^—Ba1—O14^iii^	57.43 (6)	C6—O12—Cr1	113.91 (19)
O3—Ba1—O14^iii^	152.62 (7)	C1—O13—Ba1^v^	120.99 (19)
O2—Ba1—O1	63.63 (7)	C2—O14—Ba1^v^	120.2 (2)
O17^i^—Ba1—O1	63.68 (7)	C3—O15—Ba1^ii^	119.24 (19)
O15^ii^—Ba1—O1	169.31 (7)	C4—O16—Ba1^ii^	118.81 (19)
O18—Ba1—O1	107.00 (7)	C5—O17—Ba1^i^	117.15 (19)
O16^ii^—Ba1—O1	118.91 (7)	C6—O18—Ba1	108.89 (19)
O18^i^—Ba1—O1	65.59 (7)	C6—O18—Ba1^i^	115.98 (19)
O13^iii^—Ba1—O1	124.84 (7)	Ba1—O18—Ba1^i^	121.35 (7)
O3—Ba1—O1	66.37 (8)	H19A—O19—H19B	106 (2)
O14^iii^—Ba1—O1	122.46 (7)	H20A—O20A—H20B	102 (2)
O5^iv^—Ni1—O5	180.00 (8)	H20C—O20B—H20D	103 (2)
O5^iv^—Ni1—O4^iv^	90.78 (9)	O13—C1—O7	125.2 (3)
O5—Ni1—O4^iv^	89.22 (9)	O13—C1—C2	120.2 (3)
O5^iv^—Ni1—O4	89.22 (9)	O7—C1—C2	114.6 (3)
O5—Ni1—O4	90.78 (9)	O14—C2—O8	126.5 (3)
O4^iv^—Ni1—O4	180.0	O14—C2—C1	120.2 (3)
O5^iv^—Ni1—O6^iv^	91.79 (9)	O8—C2—C1	113.3 (3)
O5—Ni1—O6^iv^	88.21 (9)	O15—C3—O9	125.8 (3)
O4^iv^—Ni1—O6^iv^	89.13 (9)	O15—C3—C4	120.3 (3)
O4—Ni1—O6^iv^	90.87 (9)	O9—C3—C4	113.9 (3)
O5^iv^—Ni1—O6	88.21 (9)	O16—C4—O10	125.4 (3)
O5—Ni1—O6	91.79 (9)	O16—C4—C3	120.6 (3)
O4^iv^—Ni1—O6	90.87 (9)	O10—C4—C3	114.1 (3)
O4—Ni1—O6	89.13 (9)	O17—C5—O11	125.6 (3)
O6^iv^—Ni1—O6	180.00 (4)	O17—C5—C6	120.2 (3)
O8—Cr1—O12	171.94 (9)	O11—C5—C6	114.3 (3)
O8—Cr1—O7	82.86 (9)	O18—C6—O12	125.1 (3)
O12—Cr1—O7	91.28 (9)	O18—C6—C5	120.1 (3)
O8—Cr1—O10	93.57 (9)	O12—C6—C5	114.8 (3)
			
Ba1^v^—O13—C1—O7	−172.7 (2)	Cr1—O10—C4—C3	−0.6 (3)
Ba1^v^—O13—C1—C2	8.4 (4)	O15—C3—C4—O16	0.9 (4)
Cr1—O7—C1—O13	−174.5 (2)	O9—C3—C4—O16	180.0 (3)
Cr1—O7—C1—C2	4.4 (3)	O15—C3—C4—O10	−178.0 (3)
Ba1^v^—O14—C2—O8	173.1 (2)	O9—C3—C4—O10	1.1 (4)
Ba1^v^—O14—C2—C1	−7.5 (4)	Ba1^i^—O17—C5—O11	−150.8 (2)
Cr1—O8—C2—O14	175.1 (3)	Ba1^i^—O17—C5—C6	27.7 (3)
Cr1—O8—C2—C1	−4.3 (3)	Cr1—O11—C5—O17	−171.9 (2)
O13—C1—C2—O14	−0.5 (5)	Cr1—O11—C5—C6	9.5 (3)
O7—C1—C2—O14	−179.5 (3)	Ba1—O18—C6—O12	20.2 (4)
O13—C1—C2—O8	178.9 (3)	Ba1^i^—O18—C6—O12	161.3 (2)
O7—C1—C2—O8	−0.1 (4)	Ba1—O18—C6—C5	−158.5 (2)
Ba1^ii^—O15—C3—O9	−166.0 (2)	Ba1^i^—O18—C6—C5	−17.4 (3)
Ba1^ii^—O15—C3—C4	13.0 (3)	Cr1—O12—C6—O18	−178.3 (2)
Cr1—O9—C3—O15	178.1 (2)	Cr1—O12—C6—C5	0.4 (3)
Cr1—O9—C3—C4	−1.0 (3)	O17—C5—C6—O18	−6.7 (4)
Ba1^ii^—O16—C4—O10	164.6 (2)	O11—C5—C6—O18	172.0 (3)
Ba1^ii^—O16—C4—C3	−14.2 (4)	O17—C5—C6—O12	174.6 (3)
Cr1—O10—C4—O16	−179.4 (3)	O11—C5—C6—O12	−6.8 (4)

Symmetry codes: (i) −*x*, −*y*+2, −*z*+2; (ii) −*x*, −*y*+1, −*z*+2; (iii) *x*−1/2, −*y*+3/2, *z*+1/2; (iv) −*x*+1, −*y*+1, −*z*+2; (v) *x*+1/2, −*y*+3/2, *z*−1/2.

### Hydrogen-bond geometry (Å, º)

**Table:**

*D*—H···*A*	*D*—H	H···*A*	*D*···*A*	*D*—H···*A*
O1—H1*A*···O16^vi^	0.88 (1)	2.20 (2)	3.038 (4)	160 (3)
O1—H1*B*···O20*A*	0.88 (1)	1.80 (2)	2.660 (18)	165 (4)
O1—H1*B*···O20*B*	0.88 (1)	2.25 (3)	3.08 (2)	156 (3)
O2—H2*A*···O10^vi^	0.88 (1)	1.98 (1)	2.851 (3)	175 (4)
O2—H2*B*···O19^vii^	0.88 (1)	1.96 (1)	2.835 (4)	175 (4)
O3—H3*A*···O7	0.89 (1)	2.32 (3)	2.993 (3)	132 (3)
O3—H3*B*···O20*A*	0.89 (1)	2.00 (2)	2.893 (16)	176 (3)
O3—H3*B*···O20*B*	0.89 (1)	1.86 (2)	2.722 (11)	163 (3)
O4—H4*A*···O3	0.88 (1)	1.96 (1)	2.819 (4)	166 (4)
O4—H4*B*···O17^vi^	0.87 (1)	1.92 (1)	2.791 (3)	179 (4)
O5—H5*A*···O11^vi^	0.87 (1)	1.96 (1)	2.811 (3)	165 (4)
O5—H5*B*···O9^iv^	0.88 (1)	1.84 (1)	2.703 (3)	167 (4)
O6—H6*A*···O13^iv^	0.87 (1)	1.91 (1)	2.761 (3)	165 (4)
O6—H6*B*···O19	0.87 (1)	1.83 (1)	2.693 (3)	172 (3)
O19—H19*A*···O1^viii^	0.87 (1)	1.94 (2)	2.759 (4)	157 (4)
O19—H19*B*···O15	0.87 (1)	1.93 (1)	2.789 (3)	172 (4)
O20*A*—H20*A*···O8^vi^	0.88 (1)	2.01 (3)	2.855 (10)	161 (8)
O20*A*—H20*B*···O20*A*^ix^	0.88 (1)	1.74 (3)	2.60 (2)	168 (9)
O20*B*—H20*C*···O8^vi^	0.88 (1)	2.11 (4)	2.893 (11)	149 (7)
O20*B*—H20*D*···O6^iv^	0.88 (1)	2.07 (4)	2.90 (3)	159 (8)

Symmetry codes: (iv) −*x*+1, −*y*+1, −*z*+2; (vi) *x*+1/2, −*y*+3/2, *z*+1/2; (vii) −*x*+1/2, *y*+1/2, −*z*+5/2; (viii) *x*, *y*−1, *z*; (ix) −*x*+1, −*y*+2, −*z*+2.
